# Task-concurrent anodal tDCS modulates bilateral plasticity in the human suprahyoid motor cortex

**DOI:** 10.3389/fnhum.2015.00370

**Published:** 2015-06-24

**Authors:** Shaofeng Zhao, Zulin Dou, Xiaomei Wei, Jin Li, Meng Dai, Yujue Wang, Qinglu Yang, Huai He

**Affiliations:** ^1^Department of Rehabilitation Medicine, The Third Affiliated Hospital, Sun Yat-sen UniversityGuangzhou, China; ^2^Department of Rehabilitation Medicine, The First Affiliated Hospital of Soochow UniversitySuzhou, China; ^3^Department of Neurology, The Third Affiliated Hospital, Sun Yat-sen UniversityGuangzhou, China

**Keywords:** brain stimulation, transcranial direct current stimulation, swallowing, plasticity, task, dysphagia

## Abstract

Transcranial direct current stimulation (tDCS) is a non-invasive method to modulate cortical excitability in humans. Here, we examined the effects of anodal tDCS on suprahyoid motor evoked potentials (MEP) when applied over the hemisphere with stronger and weaker suprahyoid/submental projections, respectively, while study participants performed a swallowing task. Thirty healthy volunteers were invited to two experimental sessions and randomly assigned to one of two different groups. While in the first group stimulation was targeted over the hemisphere with stronger suprahyoid projections, the second group received stimulation over the weaker suprahyoid projections. tDCS was applied either as anodal or sham stimulation in a random cross-over design. Suprahyoid MEPs were assessed immediately before intervention, as well as 5, 30, 60, and 90 min after discontinuation of stimulation from both the stimulated and non-stimulated contralateral hemisphere. We found that anodal tDCS (a-tDCS) had long-lasting effects on suprahyoid MEPs on the stimulated side in both groups (tDCS targeting the stronger projections: *F*_(1,14)_ = 96.2, *p* < 0.001; tDCS targeting the weaker projections: *F*_(1,14)_ = 37.45, *p* < 0.001). While MEPs did not increase when elicited from the non-targeted hemisphere after stimulation of the stronger projections (*F*_(1,14)_ = 0.69, *p* = 0.42), we found increased MEPs elicited from the non-targeted hemisphere after stimulating the weaker projections (at time points 30–90 min) (*F*_(1,14)_ = 18.26, *p* = 0.001). We conclude that anodal tDCS has differential effects on suprahyoid MEPs elicited from the targeted and non-targeted hemisphere depending on the site of stimulation. This finding may be important for the application of a-tDCS in patients with dysphagia, for example after stroke.

## Introduction

Transcranial direct current stimulation (tDCS) is a non-invasive method of neuromodulation that provides insights into functions of specific brain areas and associated motor activity (Filmer et al., 2014; Flöel, 2014). This technique involves delivering constant, weak electrical current to a local region of the cerebral cortex to modulate neuronal excitability and plasticity. tDCS is thought to elicit or inhibit brain activity through a polarity-dependent process and result in effects that persist after the stimulation has been discontinued (Nitsche and Paulus, 2001). Moreover, the changes in activity are observed both locally at the site of stimulation and distally in interconnected regions throughout the brain (Stagg et al., 2013; Liew et al., 2014).

Swallowing is an indispensable activity that requires coordination of many cranial nerves and midline muscles including those corresponding to the oral, lingual, pharyngeal, and esophageal areas. The neural network responsible for swallowing is widespread in both cortical and brainstem regions. In the past, research on the neurophysiology of swallowing was restricted to investigating the underlying mechanistic basis in the brainstem and nerves (afferent and efferent) associated with this region (Jean, 2001). However, recent advances in functional imaging and other non-invasive methods have provided an opportunity to better understand the neural basis of swallowing. Substantial evidence from neuroimaging studies has demonstrated that both the cerebral cortex and subcortical regions play an important role in controlling the swallowing process. These regions include the primary sensorimotor cortex, insula, anterior cingulate cortex, frontal operculum, and supplementary motor areas, basal ganglia, thalamus, and cerebellum (Sörös et al., 2009; Leopold and Daniels, 2010). Further, previous studies have established a distributed but functionally connected map of the neural structures involved in swallowing, which has contributed to the development of techniques that can be applied in clinical practice (Michou and Hamdy, 2009; Lowell et al., 2012; Babaei et al., 2013).

Several studies have investigated the possible application of tDCS for modulating the swallowing motor cortex in both healthy subjects and post-stroke patients with dysphagia. Jefferson et al. (2009) verified that anodal tDCS (a-tDCS) could enhance excitability of the ipsilateral pharyngeal motor cortex, as evaluated by single-pulse transcranial magnetic stimulation (TMS). Three clinical trials of post-stroke patients with dysphagia showed improvement in oropharyngeal motor function following tDCS, as assessed using clinical dysphagia scales (Kumar et al., 2011; Yang et al., 2012; Shigematsu et al., 2013). Further, magnetoencephalography (MEG) data from healthy volunteers revealed evidence of bilateral cortical activation in brain regions important for swallowing following a-tDCS over either side of the pharyngeal motor cortex (Suntrup et al., 2013). In addition, using an index to measure sucking volume and electroactivity of the suprahyoid and submental muscles, Cosentino et al. (2014) recently found that a-tDCS over the right swallowing motor cortex could increase oral sucking capacity in healthy subjects.

Despite these studies, the neural substrates underlying swallowing remain widely unknown. Unlike limb neural organization, swallowing is under bilateral, but asymmetric cortical control (Hamdy et al., 2000; Suntrup et al., 2013). Thus, dysphagia after stroke is thought to be a consequence of a lesion affecting the stronger swallowing projections, while a lesion in the weaker projections is expected to have no functional effect (Hamdy et al., 1997). Previous literature focused on the effects of a-tDCS applied over the lesioned hemisphere (Jefferson et al., 2009), while less is known about a-tDCS targeting the weaker contralesional projections. It is unknown, for instance, whether a-tDCS over weaker swallowing projections inhibits the contralateral stronger projections, similar to the findings for the upper-limb motor domain, is affected by transcallosal inhibition (Murase et al., 2004).

Furthermore, although specific stimulation parameters (e.g., stimulation dose and site and brain state during stimulation) have been shown to be decisive factors for the impact of a-tDCS on behavior (Bradnam et al., 2010; Brunoni et al., 2012; Liew et al., 2014), no study has investigated the optimal dose for a-tDCS over swallowing projections with task. In addition, the time-course of a-tDCS after-effects on swallowing are unknown.

Understanding the hemisphere-specific effects of a-tDCS targeting swallowing projections and the time-course of the after-effects can provide important insights to improve stimulation strategies for clinical practice. Therefore, this study was aimed at investigating these two issues. Specifically, we applied a-tDCS over the stronger or weaker swallowing projections concurrently with a swallowing task and investigated the effects on suprahyoid motor evoked potentials (MEP) elicited from the stimulated and non-stimulated hemisphere as well as the after-effects of a-tDCS. We hypothesized that a-tDCS applied concurrently with a swallowing task over the stronger projections would increase suprahyoid MEPs on the stimulated hemisphere only, while a-tDCS over the weaker projections would affect suprahyoid MEPs elicited from both hemispheres. We further hypothesized that the time-course of stimulation after-effects will be similar to that reported previously for tDCS effects in the motor domain.

## Materials and Methods

### Participants

A total of 37 healthy adult volunteers were initially recruited. Since no discernible suprahyoid/submental MEPs were induced in the alternative hemispheres of six subjects, they were excluded. The 31 remaining subjects were randomly divided into two experimental groups and assessed using the Edinburgh Handedness Inventory (Oldfield, 1971). One volunteer was intolerant to TMS. Therefore, 15 adults (eight men and seven women, 13 right-handed, mean ± standard deviation (SD) age: 29 ± 10 years, age range: 21–51 years) participated in the first experiment. Another 15 adults (six men and nine women, 14 right-handed, mean age: 26 ± 9 years, age range: 20–49 years) were included in the second experiment. No subject had any previous swallowing problems, had a history of neurological diseases, was pregnant, had a metal in the head or eyes, or used medication affecting the central nervous system.

Informed consent was obtained from all the subjects. The investigation was approved by the local ethics committee and conducted in compliance with the Declaration of Helsinki.

### Stimulus and Devices

#### tDCS

In each session, continuous tDCS was delivered by a battery-driven constant current stimulator (IS200, Zhineng Electronics Industrial Co., Ltd., Sichuan, China) through a pair of 4 × 6-cm rubber electrodes encased in saline-soaked sponges. The electrodes were fixed to the head with a reticular elastic cap to ensure optimal contact with the scalp. For anodal stimulation, the anodal electrode was placed over the suprahyoid/submental motor cortex, producing the largest MEPs detected by TMS, with its long axis parallel to the central sulcus, while the cathode was overlying the contralateral supraorbital ridge. Anodal conditioning was performed using a current strength of 1.5 mA, resulting in current density of 0.06 mA/cm^2^, for a duration of 20 min. These parameters were previously shown to have an optimal effect on pharyngeal motor cortex excitability and were recommended for use in clinical studies (Jefferson et al., 2009; Olma et al., 2013). For real stimulation, the current was ramped up to 1.5 mA over 15 s, eliciting a transient tingling sensation in the subjects. It was maintained for 20 min, before being slowly turned off over 15 s. During sham stimulation, the current was also ramped up over 15 s, with an equal amount of time to taper off. This blinding protocol has been demonstrated to be reliable, safe, and tolerable (Gandiga et al., 2006; Kessler et al., 2012).

#### MEP

MEPs were recorded from each suprahyoid/submental muscle group (the left and right anterior belly of the digastric, mylohyoid, and geniohyoid muscles). The recording electrode was positioned 10 mm lateral to the midline of the submental area with a 20 × 10-mm surface adhesive electrode rectangle (Sun Java Co., Ltd., Guangzhou, China). A reference electrode was mounted over the hyoid, while the ground electrode was attached to the posterior neck over the sixth cervical vertebrae spinous process (Plowman-Prine et al., 2008). All electrodes were connected to a portable electromyography and evoked potential (EMG/EP) system (NTS-2000, NCC Medical Co., Ltd., Shanghai, China) that filtered (bandpass set at 20 Hz-10 kHz), rectified, and amplified the electromyographic signal.

#### TMS

To assess corticobulbar excitability, single-pulse TMS was applied using a magnetic stimulator (CCY-I, YIRUIDE Medical Equipment Co., Ltd., Wuhan, China) with a figure-of-8 coil and an outer wing diameter of 90 mm (maximal output: 2.2 T). After marking the cranial vertex on the scalp, the optimal sites for evoking the maximum suprahyoid MEPs responses from both hemispheres were identified as “hotspots.” The area approximately 8–11 cm lateral and 0–4 cm anterior to the cranial vertex was examined to locate hotspots (Plowman-Prine et al., 2008; Doeltgen et al., 2009). Once located, the hotspots were marked using a water-soluble pen and recorded to ensure a consistent stimulation position was maintained throughout the experiments. The suprahyoid motor threshold of each hemisphere was determined using single stimulation pulses to evoke potentials of at least 50 μVon 5/10. For each subject, 10 MEPs were recorded from both hemispheres using single-pulse TMS at 120% of motor threshold; this intensity was used throughout the session.

#### Behavioral Task

Each participant was asked to accomplish 40 effortful swallows in a 20-min period. They were instructed to drink water (at room temperature), if necessary. Before experiments, all volunteers were instructed to learn how to engage in effortful swallowing, which is defined as making a conscious effort to contract their tongue and pharyngeal muscles forcefully. The swallowing task was shown using software on a computer and was carried out simultaneously with tDCS. The task was performed every 30 s with a visual and auditory cue for 2 s followed by rest for 28 s.

### Experimental Procedures

#### Experiment 1: tDCS over the Stronger Hemisphere Concurrently with the Swallowing Task

**Figure 1:**
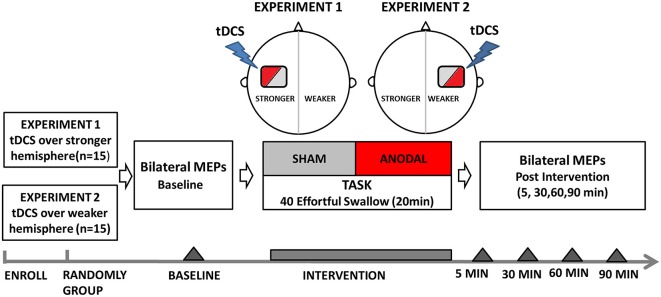
**Flow diagram of experiment protocols showing the time points for measurements and interventions**. tDCS, transcranial direct current stimulation.

Before intervention, volunteers (*n* = 15) were seated in a chair, and motor hotspots as well as thresholds for suprahyoid/submental representation were determined by single-pulse TMS (Figure 1). Baseline electromyographic data were collected from the contralateral submental region by applying a stimulus set at 120% motor threshold on both the hemispheres. The motor cortex that elicited suprahyoid MEPs of larger amplitude at the lowest threshold was defined as the stronger hemisphere (Mistry et al., 2012; Vasant et al., 2014). a-tDCS at an intensity of 1.5 mA was applied at the stronger hemisphere during the entire effortful swallowing task (20-min long). Changes in TMS-evoked suprahyoid MEPs were then measured over both (stimulated and unstimulated) hemispheres at 5, 30, 60, and 90 min post-intervention. The order of interventions (anodal and sham) was randomly assigned for each volunteer using a crossover design, and sessions were conducted at least 1 week apart to avoid any carryover effects. Two independent medical assistants performed all interventions; they were blinded to the treatment status of the subjects and research results. Subjects were blinded in a similar fashion (a double-blind protocol).

#### Experiment 2: tDCS over the Weaker Hemisphere Concurrently with the Swallowing Task

For the second experiment, another group of subjects (*n* = 15) were included (Figure 1). Single-pulse TMS was applied to locate hotspots and determine the submental motor threshold. Baseline MEP data was then obtained in the same way as in Experiment 1. The main difference from Experiment 1 is that tDCS (anodal and sham) was performed randomly on the hemisphere *contralateral* to the stronger projection (as described in Experiment 1) using a crossover design. The hemisphere that elicited weaker responses upon TMS was defined as the weaker hemisphere. Assessment of changes in bilateral TMS-evoked submental MEPs was obtained at 5, 30, 60, and 90 min post-intervention, in a similar manner to Experiment 1.

### Data Acquisition

For all experiments, amplitudes (defined as the maximum peak-to-peak value of the MEP waveform) were determined from each group of 10 electromyographic traces and then averaged. To minimize individual variability in the amplitude of suprahyoid MEPs, data were normalized to the baseline amplitude for each volunteer and expressed as a percent change from baseline.

### Statistical Analyzes

All data were analyzed using the Statistical Package for the Social Sciences (SPSS) 18.0 software (SPSS Inc., Chicago, IL, USA). A general linear model two-way repeated measure analysis of variance (ANOVA) was performed at a statistical threshold of *p* < 0.05. First, we analyzed for the variables of intervention type (anodal vs. sham tDCS) and time (baseline, 5, 30, 60, and 90 min) for each hemisphere. Using data obtained using the a-tDCS intervention, we conducted ANOVA for the variables of treatment site (stronger vs. weaker hemisphere) and time (baseline, 5, 30, 60, and 90 min). The dependent variable was defined as the percent change in MEP amplitude from baseline. Simple main effects were determined by repeated ANOVA as long as a significant interaction was present. If there was no significant interaction but only significant main effects, a *post hoc* analysis of multiple comparisons (Bonferroni correction) was used. The Greenhouse-Geisser correction was used to test for violations of sphericity, when necessary. Normalized MEP data are expressed as means [±standard error (SE)]; other data are stated as means (±SD).

## Results

### Volunteers and tDCS Impedance

A total of 30 healthy volunteers completed the trials with good tolerance for TMS and tDCS; the only exception was one subject who complained of a headache after TMS and quit. The impedance of the tDCS interventions was 5.0 ± 1.1 kΩ (±SD), with a range of 3–7.5 kΩ.

### Cortical Location and Baseline Measurements Obtained by TMS

According to the 8-figure-coil TMS location, the average distances from the cranial vertex to the hotspots of submental motor representation were as follows: left hemisphere, 9.3 ± 0.7 cm lateral, 0.7 ± 1.1 cm anterior; right hemisphere, 9.6 ± 0.8 cm lateral, 0.3 ± 0.7 cm anterior. Upon comparing the bilateral mean amplitudes of MEP traces induced by single-pulse TMS, 18 of 30 participants were observed to have a stronger suprahyoid projection in the left hemisphere, while 12 participants had a stronger right cortical representation. The average submental motor thresholds were 49 ± 3% and 53 ± 3% of the stimulation output for the stronger and weaker hemispheres, respectively.

### Experiment 1: tDCS over the Stronger Hemisphere

**Figure 2:**
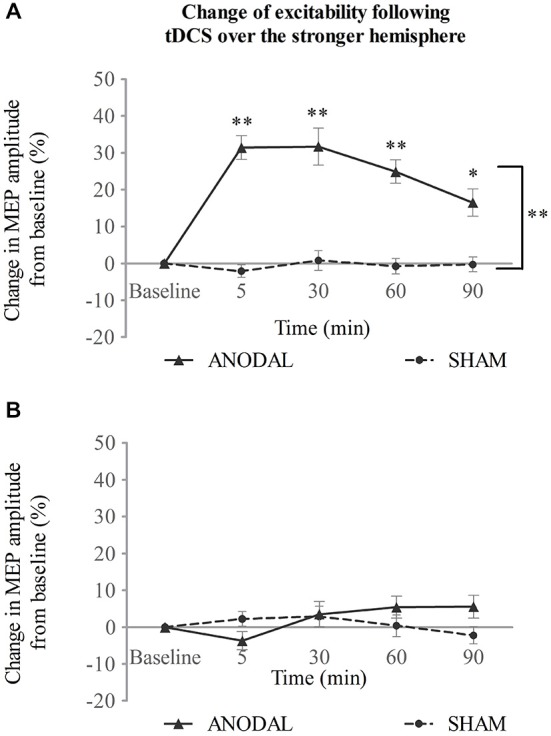
**tDCS over the stronger hemisphere concurrent with the swallowing task. (A)** a-tDCS increased suprahyoid cortical excitability in the stronger hemisphere. **(B)** a-tDCS had no effects on the weaker hemisphere (*p < 0.05; **p < 0.001, compared with sham, respectively).

When applied over the stronger submental motor cortex, a-tDCS enhanced ipsilateral excitability compared with sham (Figure 2A). Two-way repeated ANOVA revealed a significant effect for both intervention type (*F*_(1,14)_ = 96.2, *p* < 0.001) and time (*F*_(4,56)_ = 13.7, *p* < 0.001). A significant interaction was also found between intervention type and time (*F*_(3,37)_ = 11.6, *p* < 0.001). A further simple main effect analysis showed that a-tDCS resulted in increased amplitudes of MEPs compared with sham (mean difference in MEPs, 21 ± 2%; 95% confidence interval, 15–27%; *p* < 0.001). Additionally, the MEP amplitudes increased with time following a-tDCS stimulation (5, 30, and 60 min, *p* < 0.001; 90 min *p* = 0.002; Figure 2A).

No change was observed, however, for the contralateral weaker swallowing motor cortex (Figure 2B). Two-way repeated ANOVA showed no significant main effect for intervention type (*F*_(1,14)_ = 0.69, *p* = 0.42) or time (*F*_(2,31)_ = 1.35, *p* = 0.275), and no significant interaction was observed between these factors (*F*_(4,56)_ = 2.05, *p* = 0.099; see Figure 2B).

### Experiment 2: tDCS over the Weaker Hemisphere

**Figure 3:**
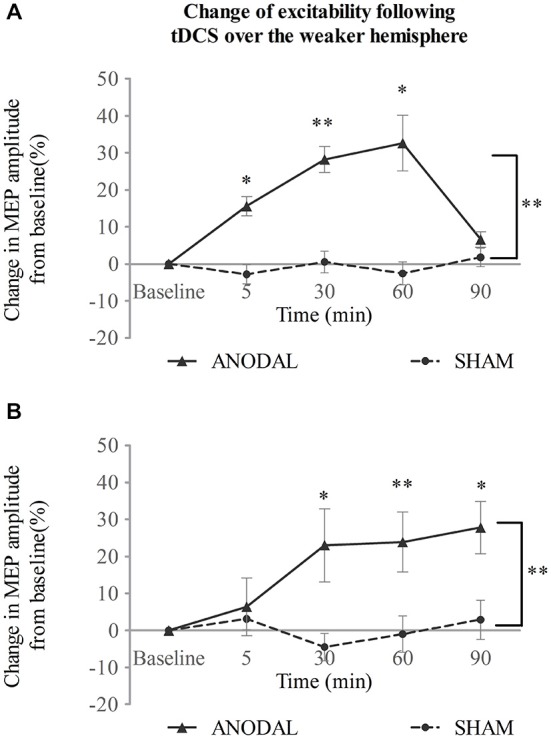
**tDCS over the weaker hemisphere concurrent with the swallowing task**. a-tDCS increases suprahyoid cortical excitability of the **(A)**weaker and **(B)** stronger hemisphere (*p < 0.05; **p < 0.001, compared with sham, respectively).

When tDCS was applied over the weaker motor cortex, excitability of the ipsilateral projection was increased after a-tDCS compared with sham intervention (Figure 3A). Two-way repeated measures ANOVA showed main effects for both intervention type and time (*F*_(1,14)_ = 37.45, *p* < 0.001; *F*_(2,32)_ = 12.15, *p* < 0.001 respectively). There was also a significant interaction between the two variables (*F*_(2,31)_ = 9.62, *p* < 0.001). A simple main effect analysis demonstrated that a-tDCS increased the MEP amplitudes compared with sham (mean difference in MEPs, 17 ± 3%; 95% confidence interval 11–23%; *p* < 0.001). Moreover, significant increase in MEPs was seen at 5, 30, and 60 min following a-tDCS (*p* = 0.001, *p* < 0.001, and *p* = 0.002 vs. sham, respectively; see Figure 3A).

In contrast to a-tDCS administered over the stronger hemisphere (Experiment 1), a-tDCS over the weaker hemisphere facilitated contralateral excitability as well (Figure 3B). A significant main effect was observed for invention type and a significant interaction was observed between variables (*F*_(1,14)_ = 18.26, *p* = 0.001; *F*_(2,34)_ = 3.91, *p* = 0.023, respectively; two-way repeated measures ANOVA), but no main effect was observed for time (*F*_(2,22)_ = 2.13, *p* = 0.151). A simple main effects analysis showed that a-tDCS enhanced MEP amplitudes (mean difference in MEPs, 16 ± 4%; 95% confidence interval, 824%; *p* = 0.001 vs. sham). Compared to sham, a-tDCS, increased MEP amplitudes at 30, 60, and 90 min following stimulation (*p* = 0.009, *p* < 0.001 and *p* = 0.007, respectively; see Figure 3B).

### Effects of a-tDCS over the Bilateral Hemispheres

**Figure 4:**
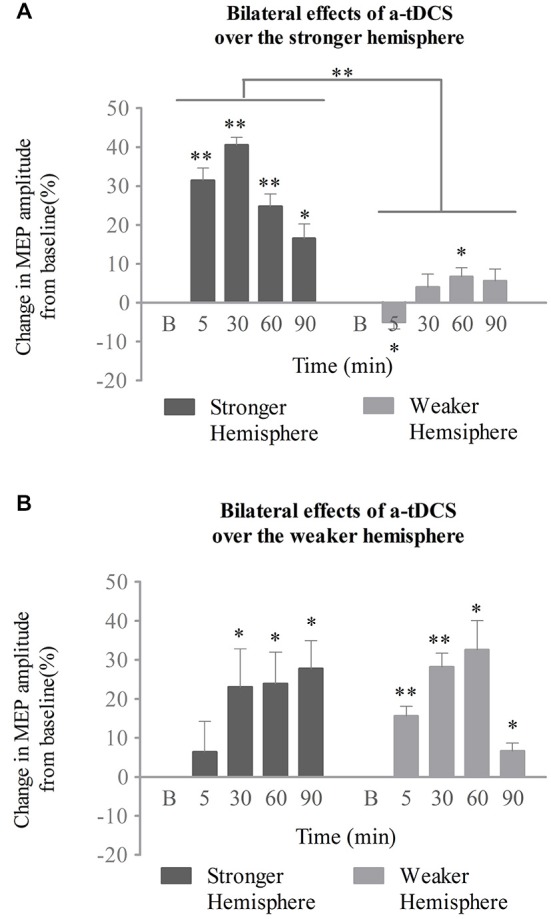
**Effects of a-tDCS over bilateral neurophysiology concurrent with the swallowing task. (A)** When the stronger hemisphere was targeted, a-tDCS enhanced ipsilateral, but not contralateral, suprahyoid region cortical excitability. The peak response of MEPs on the stronger hemisphere was observed at 30 min post a-tDCS. **(B)** When the weaker hemisphere was targeted, a-tDCS enhanced both ipsilateral and contralateral suprahyoid-region cortical excitability. The peak response of MEPs on the stronger hemisphere was observed at 90 min, while that on the weaker hemisphere was present at 60 min post a-tDCS (*p < 0.05; **p < 0.001, compared with baseline, respectively).

In the stronger cortex stimulation group, we also tested the effects of a-tDCS intervention on both the stimulated (stronger) and unstimulated (weaker) hemispheres. Two-way repeated ANOVA with stimulation site (stronger vs. weaker) and time (baseline, 5, 30, 60, and 90 min post-intervention) confirmed significant main effects for both (*F*_(1,14)_ = 306.25, *p* < 0.001; *F*_(2,29)_ = 23.84, *p* < 0.001, respectively), as well as an interaction between the two variables (*F*_(2,33)_ = 19.58, *p* < 0.001; see Figure 4A). As determined by a simple main effects analysis of time, excitability of the stronger cortex was found to be significantly increased after stimulation compared to baseline (5, 30, and 60 min, *p* < 0.001; 90 min, *p* = 0.01, respectively). The effect reached its peak at 30 min post-stimulation (mean MEPs, 41 ± 2%; 95% confidence interval, 37–45%; *p* < 0.001). However, the MEP amplitudes from the weaker cortex fluctuated across the baseline after stimulation (5 min, *p* = 0.017; 30 min, *p* = 0.242; 60 min, *p* = 0.011; 90 min, *p* = 0.093; see Figure 4A). The interaction was therefore attributed to an increase in excitability of the stronger submental cortex.

The above interactions were not found in the weaker cortex stimulation group. A two-way repeated ANOVA analysis revealed a significant main effect only for time (*F*_(2,27)_ = 8.5, *p* = 0.002). Neither a significant main effect for stimulation site (*F*_(1,14)_ = 0.01, *p* = 0.914) nor any interaction among variables (*F*_(2,30)_ = 2.91, *p* = 0.068) was observed. Following *post hoc* analysis, significant enhancements of MEPs from the stronger (unstimulated) projection were observed at 30, 60, and 90 min compared with baseline (*p* = 0.035, *p* = 0.011, *p* = 0.002, respectively); the effect peaked at 90 min (mean of MEPs, 28 ± 7%; 95% confidence interval, 12–43%; *p* = 0.002). Excitation of the weaker (stimulated) projection was enhanced after stimulation for all time points measured (5 and 30 min, *p* < 0.001; 60 min, *p* = 0.001; 90 min, *p* = 0.007, respectively), and reached its peak at 60 min (mean of MEPs, 32 ± 8%; 95% confidence interval, 16–49%; *p* = 0.002; see Figure 4B).

## Discussion

Our study investigated bilateral plasticity of the suprahyoid motor cortex in response to task-concurrent a-tDCS on the stronger and weaker hemispheres. We demonstrated that a-tDCS on the stronger hemisphere could only increase excitability of the ipsilateral submental motor cortex. However, when applied on the weaker hemisphere, a-tDCS could enhance both ipsilateral and contralateral excitation. These findings imply that a-tDCS modulates suprahyoid motor representation and the associated neural plasticity in a site-dependent manner, which merits further discussion.

Previous studies on tDCS of the swallowing motor cortex found that anodal stimulation could improve behavior and excitability of corticobulbar projections (Jefferson et al., 2009; Shigematsu et al., 2013). However, the parameters for optimal efficacy, such as intensity and duration of stimulation, are still poorly defined. One experiment showed that only a high intensity (1.5 mA) or long duration (20 min) of a-tDCS can produce activity effects on the pharyngeal cortex (Jefferson et al., 2009). Another pilot investigation employed a different protocol (2 mA for 30 min) for tDCS of the swallowing motor area and demonstrated a therapeutic benefit in stroke patients with dysphagia (Kumar et al., 2011). However, the increased intensity and duration protocols examined in other studies of non-invasive brain stimulation did not always induce a strong response in the motor cortex, and in some cases even had the reverse effect (Hummel et al., 2005; Gentner et al., 2008). We expected that an a-tDCS intensity of 1.5 mA for duration of 20 min (with a current density of 0.06 mA/cm^2^) would enhance corticobulbar excitability in both local and remote cortical areas. Interestingly, excitability of the stimulated hemisphere was upregulated regardless of which hemisphere (stronger or weaker) received the intervention. These results indicate that the parameters of a-tDCS applied in our study did not reach a ceiling effect, and could therefore facilitate excitation of the suprahyoid motor cortex.

Importantly, the observation of site-dependent plasticity in the submental motor cortex implies that during difficult tasks, a-tDCS of the weaker, and not stronger, submental projection more readily facilitates activation of the contralateral motor system. These effects can be explained by the interhemispheric theory (Bloom and Hynd, 2005), which assumes that the interaction between the two hemispheres is a dynamic process and can be flexibly modulated by either task or exogenous stimulation (Murase et al., 2004; Silvanto et al., 2009). Contrary to the interhemispheric competition observed in the hand motor system (Ferbert et al., 1992; Meyer et al., 1998; Daskalakis et al., 2002) and other cognitive processing domains (Silvanto et al., 2009; Chrysikou and Hamilton, 2011), the bilateral swallowing motor cortices, such as those corresponding to the pharyngeal region, work synergistically. This hypothesis was first postulated from longitudinal observations in unilateral stroke patients with dysphagia (Hamdy et al., 1998). Evidence from neuroimaging and electrophysiology studies, both in intact projections and conditioned hemispheres, also support this model (Mistry et al., 2007; Suntrup et al., 2013). However, the first tDCS study focusing on the pharyngeal motor cortex indicated that MEP amplitude was not affected by a-tDCS over the opposite hemisphere (Jefferson et al., 2009). Our data also indicated no stimulation-dependent difference in contralateral excitability as measured by suprahyoid MEP amplitude elicited by tDCS of the stronger hemisphere. This finding suggests that increased cortical excitability of weaker projections can extend through transcallosal transmission to the stronger projections, but not vice-versa, possibly because of inhibitory synaptic mechanisms. However, differences can occur if the brain-state is disrupted by injury or conditioning, differences can occur. For instance, administering a-tDCS results in increases in excitabilities of both pharyngeal projections when preconditioning with unilateral inhibitory repetitive TMS has been conducted (Vasant et al., 2014). Other non-invasive brain stimulation studies showed that 5 Hz TMS also increased excitability of stimulated- and unstimulated-pharyngeal projections (Gow et al., 2004). In contrast, in the context of the effortful swallowing task used in our study, a-tDCS applied to the weaker projection was more likely to produce interhemispheric collaboration than the same treatment on the stronger projection. This factor may help inform clinical decisions regarding the optimal choice of target hemisphere. For example, our findings indicate that a-tDCS applied over the contralesional hemisphere of stroke patients with dysphagia might be an effective strategy to increase bilateral excitability of the suprahyoid motor cortex and improve swallowing.

The discrepancy in excitability between the stronger and weaker hemispheres in response to non-invasive brain stimulation of the swallowing cortical network has also been reported by other groups. In a TMS study, excitation of the weaker hemisphere was enhanced when intermittent theta burst stimulation was applied to the stronger pharyngeal projection (Mistry et al., 2012). However, that applied to the weaker projection resulted in no changes in excitability for either hemisphere. Apart from the use of the stronger hemisphere hypothesis to explain the brain processes underlying swallowing, another view of cortical lateralization suggests that the underlying neural substrates for this behavior are differentially lateralized. This hypothesis suggests that the left hemisphere controls the oral phase and volitional components, while the right hemisphere may be responsible for the pharyngeal phase and reflective process (Daniels et al., 2006; Teismann et al., 2009). Although there are different explanations of lateralization in swallowing, all are consistent with the hypothesis that a pattern of bilateral but asymmetric control describes the underlying cortical organization.

As a novel non-invasive brain stimulation technique, tDCS has the advantage of being highly accessible for both treatment and research. Neurophysiological studies investigating tDCS have suggested that it modulates cortical excitability in a polarity-dependent pattern (Nitsche and Paulus, 2001). The anode increases, whereas the cathode decreases, neuronal activity through a shift in resting membrane potentials. Pharmacological studies suggest that the conductance of sodium and calcium channels, activity of *N*-methyl-D-aspartate receptors, and brain-derived neurotrophic factor signaling determine the after-effects of a-tDCS (Nitsche et al., 2003; Fritsch et al., 2010). Neuroimaging studies utilizing electroencephalography, functional magnetic resonance imaging, and MEG provide evidence that tDCS also induces changes in connectivity in the neural network of the motor system (Polanía et al., 2011a; Pellicciari et al., 2013). Most importantly, tDCS paired with an active behavioral task was found to augment the network’s activity (Fritsch et al., 2010). In a series of functional magnetic resonance imaging studies, a-tDCS over the region of M1 corresponding to hand control significantly increased functional connections to the premotor and superior parietal regions (Polanía et al., 2011b). Such stimulation also facilitated connections to subcortical structures on the ipsilateral hemisphere, such as the thalamus (Polanía et al., 2012). We therefore speculated that a-tDCS might activate the contralateral suprahyoid motor region by modulating cortical and subcortical connections within the swallowing network. Future studies are needed to test this speculation.

A few limitations of the current study should be noted. Firstly, we did not examine the influence of different tDCS intensities and durations while subjects were performing the task. Considering that a previous research has determined the optimal parameters of tDCS over the pharyngeal motor cortex in the absence of a task (Jefferson et al., 2009), we combined these parameters with clinical protocols and provided evidence that more excitation occurs following tDCS concurrent with a task. Secondly, we did not apply cathodal stimulation as our study aimed for investigating stimulation strategies that would improve swallowing behavior in patient populations and previous studies suggested that cathodal tDCS over the pharyngeal motor cortex inhibits ipsilateral activity with no effect on swallowing (Jefferson et al., 2009; Cosentino et al., 2014). It cannot be excluded, though, that cathodal tDCS over the stronger projections could disinhibit activity of weaker projections, an issue that needs further investigation. Next, we did not investigate the effects of sham tDCS without a task. However, our sham group data have indirectly demonstrated that no effect on excitability following the task compared with baseline. Although task-induced changes in cortical excitability appear to be related to the degree of complexity and intensity of the task (Perez et al., 2004), there is no clear consensus regarding how this kind of swallowing behavior affects excitability (Gallas et al., 2009; Al-Toubi et al., 2011). A future study on different types and timing of swallowing-related tasks with tDCS would help elucidate the specific role of motor learning in this context.

It should also be mentioned that we did not assess the tDCS-induced changes in behavioral characteristics by means of videofluoroscopy or electromyography (Cosentino et al., 2014; Vasant et al., 2014). Therefore, we can only infer that the improvement in swallowing function could be the result of facilitative a-tDCS with task training. Nevertheless, the complex pathology of dysphagia is attributable not only to weakness of the muscles involved in swallowing, but also to a lack of coordination of the oral and pharyngeal phases. Considering the non-linear relationship between excitability and swallowing motor function in patients with dysphagia, caution is advised regarding the clinical application of this treatment protocol until double-blinded, randomized clinical trials have been completed.

In summary, we demonstrated that a-tDCS concurrent with a swallowing task can have beneficial effects on the neurophysiology controlling swallowing behavior. Moreover, we showed that these facilitations occur in a site-dependent manner. Task-concurrent a-tDCS on the weaker hemisphere not only increases excitation of the ipsilateral swallowing region, but also facilitates activation of the contralateral motor cortex. These results are of clinical relevance and confirm that a-tDCS applied over an undamaged hemisphere combined with a task appears to be an effective and safe method to treat patients with post-stroke dysphagia (Kumar et al., 2011). However, further work is needed to explore the behavioral benefits resulting from different types of tasks and durations of tDCS.

## Author Contributions

SZ performed the experiments, analyzed the data, and wrote the manuscript. ZD supervised the experiments and revised the manuscript; XW assisted with data analysis and explanation. JL and MD helped to perform the experiments and analyze the data. YW and QY recruited subjects and performed blinded treatments, and HH helped with writing the manuscript.

## Conflict of Interest Statement

The authors declare that the research was conducted in the absence of any commercial or financial relationships that could be construed as a potential conflict of interest.

## Abbreviations

tDCS: transcranial direct current stimulation
a-tDCS: anodal transcranial direct current stimulation
TMS: transcranial magnetic stimulation
MEP: motor-evoked potentials
MEG: magnetoencephalography.
